# Kombucha Bacterial Cellulose: A Promising Biopolymer for Advanced Food and Nonfood Applications

**DOI:** 10.3390/foods14050738

**Published:** 2025-02-21

**Authors:** Talita Ribeiro Gagliardi, Adriana de Farias Nascimento, Germán Ayala Valencia

**Affiliations:** 1 Laboratory of Marine Shrimp, Department of Aquaculture, Federal University of Santa Catarina, Florianópolis 88040-900, SC, Brazil; talitagagliardi@gmail.com; 2 Department of Chemical and Food Engineering, Federal University of Santa Catarina, Florianópolis 88040-900, SC, Brazil; eng.nascimentoadriana@gmail.com

**Keywords:** bacterial cellulose, kombucha, macromolecule, food ingredient, packaging material, pellicle, SCOBY

## Abstract

The pellicle is a coproduct of kombucha beverage production without economic value. This material is based on cellulose produced from bacteria and has better physical properties than cellulose isolated from plants. This review systematically analyzed the research literature about pellicle (KBC—kombucha bacteria cellulose) valorization. In general, KBC has been used in food applications, especially as a packaging ingredient to improve the physical properties of biopolymer-based films, as well as to manufacture packaging materials based on KBC. In addition, some studies have investigated the potential of KBC to encapsulate food ingredients or as a food additive. Furthermore, KBC has been used in nonfood applications with a special interest in the development of materials for textile and medical applications and as a substitute for disposable materials (e.g., spoons). Although the literature shows promising results, it is necessary to increase the production scale of this material, as well as to analyze its economic viability. It is also necessary to establish quality standards and international regulations for KBC with respect to its different food and nonfood applications.

## 1. Introduction

Kombucha is a carbonated beverage produced by tea fermentation from a symbiosis of bacteria and yeasts. This beverage has a slightly sweet and citrusy taste, with low alcohol content and a vinegar-like taste [1]. Kombucha was originally consumed in China, but this product has recently been commercialized worldwide due to the increase in the market and the potential health benefits associated with the presence of bioactive molecules and probiotics [2,3,4].

**Figure 1:**
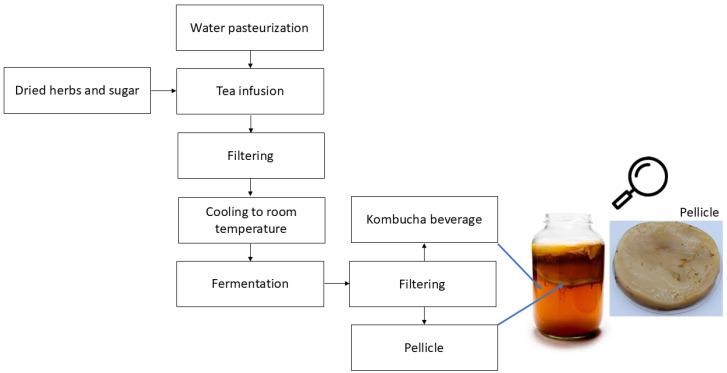
Kombucha production flowchart.

The kombucha production process involves two steps. First, a sweet tea is produced with a filtered herbal infusion to which an inoculum is added (Figure 1). The fermentation time oscillates between 7 and 15 days, depending on temperature (18–26 °C) [5]. Ethanol, acetic, lactic, glucuronic acids, as well as a large quantity of tea-derived polyphenols, are the most common products found in kombucha tea after fermentation [6,7]. In sequence, the fermented beverage in step 1 is blended with fruit juice or another herbal infusion to flavor kombucha (Figure 1). The resulting blended beverage is packed and again fermented under the same conditions. After this second step, a flavored kombucha is obtained and commercialized under refrigeration [8].

Another product of kombucha fermentation is the pellicle (Kombucha Bacterial Cellulose—KBC; Figure 1) produced by the microbial consortium, which consists of pure cellulose fibrils [9,10]. Some researchers have focused on the characterization and potential applications of KBC, probably because kombucha tea has a high economic value and KBC is considered a residue [11]. However, KBC does not contain hemicellulose and lignin, and has higher purity, degree of polymerization, and crystallinity than cellulose isolated from plants. Furthermore, materials based on KBC have better mechanical strength, water-holding capacity, chemical stability, and biological adaptability than their counterparts using plant cellulose [5,12]. In addition, the processes to purify plant cellulose demand high energy consumption and have negative impacts on cellulose chains (polymer degradation) and the environment because chemical residues are discarded during the processes [13]. In this scenario, the valorization of KBC involving a circular economy concept is fundamental to the development of a sustainable industry to achieve the 2030 Agenda for Sustainable Development, adopted by all United Nations, and focuses on the 17 Sustainable Development Goals (SDGs) [14].

Previously, Laavanya et al. [5] reviewed the KBC production, its biochemical composition and explored some potential applications. It should, however, be noted that, to our current knowledge, no review paper has comprehensively analyzed and reviewed the recent advances in KBC applications. Therefore, the objective of this review was to comprehensively analyze the potential of KBC for industrial applications. 

## 2. Research About Kombucha Bacterial Cellulose

**Figure 2:**
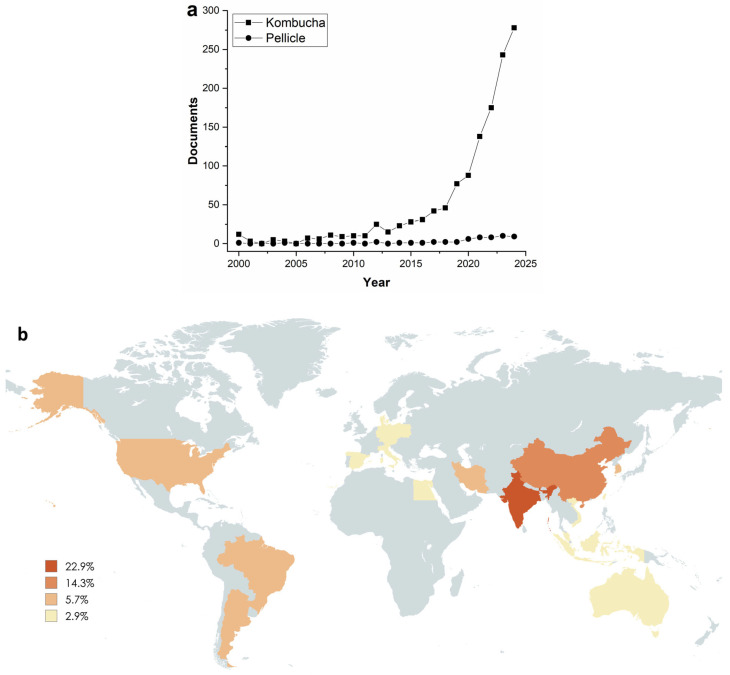
(**a**) A search of the SCOPUS database for scientific production using the keywords “kombucha” versus “kombucha AND pellicle AND scoby” between the years 2000 and 2024; (**b**) global production of scientific articles on kombucha bacterial cellulose. The world map was created using MapChart (https://www.mapchart.net/, accessed on 2 February 2025).

A search using the SCOPUS database revealed that the number of scientific manuscripts on “kombucha” has increased in the last 24 years (1287 documents) (Figure 2a). A new search using the same database and the keywords “kombucha AND pellicle AND scoby” revealed 51 research papers from the last 24 years (Figure 2a). Of these, only 26 manuscripts focused on the valorization and application of the pellicle obtained after kombucha production. These results confirmed that research on kombucha is an important topic with an increasing publication rate; however, pellicle from kombucha has been little explored.

Analyzing the global production of scientific articles on KBC, it is possible to conclude that several countries are researching this topic, with India as the country that most research about BKC (22.9%), followed by China (14.3%), Argentina, Brazil, Iran, South Korea, and USA (5.7 5 each), and Austria, Australia, Czech Republic, Denmark, Egypt, Germany, Italy, Indonesia, Malaysia, Poland, Spain, Taiwan, and Vietnam (2.9% each) (Figure 2b).

## 3. Application of Kombucha Bacterial Cellulose

**Figure 3:**
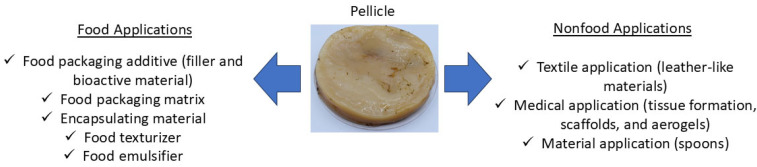
Main food and nonfood kombucha bacterial cellulose applications.

KBC has potential food and nonfood applications (Figure 3). In food, this macromolecule has been used to manufacture food packaging, as well as encapsulating material, and as a food texturizer and emulsifier. Some nonfood applications involve using KBC to manufacture leather-like materials, spoons, scaffolds, and as support to tissue formation and aerogels (Figure 3).

### 3.1. Packaging Materials

Packaging is important for the food processing, preservation, supply, and distribution chain. Its main function is to contain and protect food material, but it is also essential to ensure food quality and safety, while also extending shelf life and minimizing food loss and waste [15,16].

KBC has been used as an additive or as the main macromolecule to manufacture food packaging materials (Figure 3). The drying method affects the physicochemical properties of KBC and its application as a packaging material. Dey et al. [17] investigated the effects of different drying methods on the physicochemical properties of KBC. In this study, KBC was washed with distilled water and then soaked with NaOH (0.1 N) to remove tannins and microbial residues. In sequence, the cleaned KBC was dried using microwave drying (180–900 W), hot air oven drying (30–70 °C), and shade drying (25 °C). The authors observed that the elevated temperatures and power levels during the drying processes increased the formation of the inter-fiber hydrogen bonding of KBC. Therefore, the elastic modulus (EM) increased from 980 to 1972 MPa as the power level increased from 180 to 900 W (microwave drying) and from 529 to 1518 MPa as the drying temperature increased from 30 °C to 70 °C in oven drying. In contrast, KBC with higher water absorption (≈55%) values were obtained when this material was dried in an oven at 30 °C or in shade. Packaging materials must have high mechanical parameters and low water sensitivity. This research demonstrated that KBC must be dried using drying technologies (microwave and hot air oven drying) at elevated power levels and temperatures to produce dried KBC with high EM and less water absorption.

In another research, Ramírez Tapias et al. [18] studied the production of KBC (pellicle) using different herbs (black tea, green tea, yerba mate, Patagonian lavender, oregano, and fennel) and concluded that in all cultures, pellicle production resulted in a maximum after 21 days. Native pellicles from yerba mate had a remarkable antioxidant activity of 93 ± 4% of radical inhibition due to plant polyphenols, which could prevent food oxidation. Results revealed that the pellicles retained natural bioactive substances, preserving important physicochemical properties, which are essential for developing active materials in packaging applications.

Table 1 summarizes the use of KBC in packaging applications. Most studies have used KBC as a filler to reinform biopolymer-based films manufactured by casting and extrusion (Figure 3). In these studies, the use of KBC improved the mechanical, optical, and barrier properties and imparted antioxidant and antimicrobial activities to the developed materials (Table 1). However, this macromolecule can also be used as a polymeric matrix to manufacture packaging materials or as an encapsulating material of bioactive molecules (e.g., anthocyanins and essential oils) in packaging applications (Table 1 and Figure 3).

### 3.2. Food Applications

Pickering emulsions use solid particles to stabilize the interface between the two immiscible liquids, thereby avoiding the use of conventional surfactants [28,29]. Recently, KBC (0.5–2% *w*/*v*) was used to stabilize O/W (20:80) emulsions where this biopolymer had reduced lipid oxidation and increased emulsion stability until 96 h at 20 °C [28,30].

Finally, KBC has been used as an additive in mango jam. The incorporation of KBC between 20 and 100 g/kg reduced the water activity by up to 22.2% (0.68), moisture content from 37.1% to 19.9%, and pH from 5.9 to 3.2. Furthermore, the texture of the jam with KBC gave higher gel strength and adhesiveness. Overall acceptability in sensory test scoring was above 70% on a nine-point hedonic scale with the 40 g/kg KBC jam chosen as the most preferred [31].

### 3.3. Other Material Applications

KBC-based materials have potential nonfood applications (Figure 3). KBC has been used to develop materials with promising industrial applications. In this way, KBC was incorporated with gold nanoparticles (AuNP), silver nanoparticles (AgNP), and graphene oxide (GO). The incorporation of nanomaterials increased the glass transition (Tg = 41 → 50 °C), tensile strength (TS 63 → 94.1 MPa), and improved the hydrophobicity (WCA = 31 → 81°), probably by the formation of non-covalent interactions. Furthermore, the presence of AgNP inhibited the growth of *S. aureus*. However, whether the nanomaterial concentration was added to KBC is not known. The authors concluded that this nanocomposite can be used as a leather-like material with antimicrobial activity [32].

In another study, Candra et al. [33] synthesized AuNPs using a composite matrix based on chitosan and KBC. The obtained nanocomposite displayed an antimicrobial effect against *Pseudomonas aeruginosa* and *Streptococcus mutans*, as well as in vivo wound healing in mice, encompassing granulation tissue formation, reepithelization, reduced inflammation, and collagen fiber formation. Based on these results, KBC incorporated with AuNPs was a suitable candidate for medical applications.

Plastic spoons contribute significantly to environmental problems because they are typically made from non-degradable polymers. With this in mind, Muralidharan et al. [34] developed a multi-layered composite spoon based on KBC and gelatin. KBC was cleaned with NaOH (0.5 N) at 90 °C for 1 h, followed by distilled water until pH 7. Bilayer materials were produced by the layer-by-layer method where KBC and gelatin sheets were hot-pressed under a pressure of 2500 psi for 5 min at 90 °C. The resulting materials had a high tensile strength of 47.7 MPa and a flexural strength of 117.27 MPa, indicating high mechanical stiffness with the lowest density value of 0.71 g/cm^3^. In another research, Nguyen et al. [35] modified KBC with different silanes (dimethyldichlorosilane, hexadecyltrimethoxysilane, vinyltriethoxysilane, and 3-aminopropyltriethoxysilane) followed by mixing with polyurethane (PU) and polylactic acid (PLA). According to the authors, the optimal biocomposite formulation was KBC 13.74% *w*/*w*, PU 73.89% *w*/*w*, and PLA 12.50% *w*/*w* compressed at 155 °C for 5 min. The KBC modification with silanes increased the WCA from 82° to 95° and reduced the water permeation through the material. In this way, WCA after 5 min was 63° and 92° in untreated and treated KBC, respectively. Results from both studies indicated that disposable materials can be manufactured using KBC as the matrix.

Aerogels are nanostructured materials with low bulk density and open porosity. Recently, they have been broadly investigated because of their capacity to load vitamins, chemical compounds, and oils for food and material applications [36]. Aerogels based on KBC were produced after the purification of KBC with KOH, followed by acidic hydrolysis (H_2_SO_4_ and HCl) and lyophilization. The obtained nanofibers presented a thickness of between 50 and 110 nm with high crystallinity (90%) [37]. KBC aerogels have also been applied to 3D bioprinting in tissue engineering [38] and as acoustic foams [39].

Finally, KBC is a suitable material to create biocompatible scaffolds for tissue engineering. Recently, Rzhepakovsky et al. [40] produced a scaffold based on KBC and gelatin and dried it using lyophilization. The KBC incorporation increased the scaffold solubility and biocompatibility. These materials were implanted in rats, observing the low immunogenicity and intense formation of collagen fibers, as well as the active germination of new blood vessels. The authors concluded that KBC is an efficient macromolecule for producing materials with medical applications [40].

## 4. Future Trends

### 4.1. Cellulose Production

Cellulose is considered the most abundant renewable macromolecule available worldwide. This macromolecule is a polymer of glucose (C_6_H_12_O_6_), possessing a rotation (≈180°) of one glucose molecule in relation to the next glucose to form *β* (1–4)-linked residues, and is known as cellobiose (C_12_H_22_O_11_) [41]. In nature, approximately 1011–1012 t are synthesized annually in a pure form in the cell wall of plants by photosynthesis [42]. Wood and cotton are the main commercial sources of commercial cellulose due to their high cellulose concentrations. However, this valuable macromolecule is combined with lignin and hemicelluloses, making it necessary to isolate cellulose from the cell walls of woody plants [43]. Cellulose production from plants faces two major challenges. The first is associated with the excessive use of land and its deterioration through monocultures; the second is related to the chemical reagents used to isolate cellulose from the cell walls [41,43]. The production of KBC is key to producing cellulose involving the circular economy and sustainability concepts. A limited number of studies have investigated the physicochemical properties of KBC. However, it has been reported that KBC is finer and more unbranched than plant cellulose, which imparts better mechanical properties in the resulting materials [5]. In this way, future studies must investigate the chemical structure, crystallinity, molecular mass, and purity of KBC and the impact of fermentation process on these properties, as well as scaling up the production of KBC to an industrial scale.

### 4.2. Food and Pharmaceutical Applications

Future research should investigate the biodegradability and toxicity of KBC. Regarding toxicity, both kombucha tea and scoby are not toxic when produced under controlled conditions and are considered safe for consumption and use [44]. However, they should be used in moderation due to contraindications in infants; children under 4 years old; patients with renal, hepatic, and pulmonary diseases; and individuals with HIV. Furthermore, this product is not recommended for pregnant women due to the interference with the coagulation process, which may be harmful to fetal development. According to the literature, continuous consumption above 355 mL may cause internal organ perforation, resulting in renal lesions and necrosis in the duodenum, pancreas, and intestines [1]. The effects associated with kombucha consumption are still unclear, requiring further investigations. Currently, the regulations in effect for kombucha production address criteria such as pH and alcohol content to ensure food safety and quality for consumers.

Kombucha production conditions, such as the type of tea used, the fermentation time, and the microbial composition of the scoby are determining factors in its physicochemical, microbiological, and bioactive properties [45,46]. There is no doubt that both kombucha and scoby contain significant bioactive compounds, including polyphenols, organic acids, amino acids, vitamins, and minerals [1]. However, it is necessary to identify and quantify the viable microorganisms in KBC because this product can be used as a source of probiotics [1]. According to the literature, the use of green tea results in a quantitatively richer microbial composition, with free amino acids, reducing sugars, and increased antiproliferative activity against cancer cell lines, attributed to the presence of catechins [46,47,48]. Kombucha based on black tea, in turn, has demonstrated greater diversity and abundance of phenolic compounds, resulting in superior antioxidant capacity, presenting 70.2% flavonoids, 18.3% phenolic acids, 8.4% other polyphenols, 2.3% lignans, and 0.8% stilbenes [47]. These findings highlight that the choice of tea significantly influences the antioxidant properties of kombucha and scoby, indicating the need to study blends and the addition of new unconventional herbs to kombucha to evaluate their overall properties.

### 4.3. Animal Feed

In the animal feed industry, both kombucha and KBC have emerged as potential functional additives. Their biochemical composition, which is rich in probiotics, organic acids, and bioactive compounds, suggests significant benefits for animal nutrition, especially in the context of the growing demand for sustainable alternatives that repurpose organic waste [8].

Studies have already demonstrated these benefits in different species. Ramadhan et al. [48] supplemented the diet of catfish (*Clarias* sp.) with kombucha and observed a positive impact on growth, with increased weight gain, improved absolute growth rate, and better feed efficiency. These findings indicate that kombucha and KBC could be viable alternatives in formulating feed for aquatic animals. For terrestrial animals, Afsharmanesh and Sadaghi [49] investigated the use of probiotics, including kombucha, in the diet of broiler chickens. The results were promising: fermented tea exhibited growth-promoting effects comparable to those traditional antibiotics, reinforcing its potential as a natural alternative in poultry farming.

In addition to lactic acid bacteria, yeasts play a fundamental role in KBC, actively participating in the fermentation of kombucha. In this context, some fungi have been studied as nutritional supplements due to their probiotic and enzymatic potential. Singh et al. [50] investigated the supplementation of white button mushrooms (*Agaricus bisporus*) in the diet of *Penaeus vannamei* (Pacific white shrimp) and observed a significant improvement in the survival rate, specific growth rate, feed conversion efficiency, and protein efficiency ratio, along with increased average weight gain. These findings suggest that the presence of beneficial microorganisms in animal feed can contribute to better performance and improved nutrient absorption efficiency.

The probiotics present in kombucha play a crucial role in modulating the intestinal microbiota. Research indicates that its consumption can help balance the gut microbiota in animals, promoting better digestibility and absorption of essential nutrients [51]. In addition, antioxidant compounds such as polyphenols help reduce oxidative stress, strengthen the immune system, and increase resistance to infections, which directly impacts the physiological performance of animals [52]. These benefits, combined with the fact that the KBC, a by-product of kombucha fermentation, can be repurposed as an ingredient in animal feed, make it a promising alternative aligned with the global trend toward more sustainable and natural practices in animal nutrition [53].

Despite its great potential, more studies are still needed to validate the efficacy of kombucha on a large scale. The composition of the beverage can vary considerably due to factors such as the fermentation time and the ingredients used, making standardization and the determination of ideal dosages challenging. However, the growing interest in sustainable solutions and the search for alternatives to synthetic antibiotics and additives, kombucha is likely to gain more visibility in research and the animal feed industry. Its potential to enhance animal performance and reduce environmental impact makes it a strong candidate to establish itself as a viable functional feed additive for the future of animal nutrition.

### 4.4. Nonfood Applications

As discussed in Section 3.3, some studies have explored the use of KBC in textile, medical, and material applications. It is necessary to explore new KBC applications in these fields, such as in the production of porous materials to be used as scaffolds, adsorbents, membranes, and conductive materials, as well as for 3D printing materials, and modified cellulose such as cellulose ether, methyl cellulose, carboxymethyl cellulose, ethyl cellulose, hydroxyethyl cellulose, hydroxypropyl cellulose, cellulose ester, cellulose acetate, cellulose nitrate, and cellulose sulfate [54]. Furthermore, this macromolecule can be used to manufacture disposable plates and packaging for nonfood applications. However, before an industrial application, it is necessary to establish regulatory frameworks with the intention of standardizing the physicochemical properties of this macromolecule and its production at an industrial scale.

## 5. Conclusions

Pellicle or kombucha bacterial cellulose (KBC) is a coproduct of the kombucha beverage production without economic value. Recent studies demonstrate that KBC has potential food and nonfood applications. KBC is composed of cellulose of high purity and has been used as an encapsulating material, food texturizer, and food emulsifier. Furthermore, this macromolecule can be used to encapsulate the active molecules in functional foods. Other applications range from the production of packaging, leather-like materials, spoons, scaffolds, and as support to tissue formation and aerogels. Future studies must standardize KBC production on an industrial scale and establish international legislation regarding KBC. New KBC applications are expected to be explored because this material has antioxidant properties and can be used to produce functional foods and feeds or to manufacture active materials. Furthermore, KBC can be used to produce nanocellulose and porous materials for culture meat and adsorbents.

## Figures and Tables

**Table 1:** Application of kombucha bacterial cellulose (KBC) in food packaging materials.

Polymeric Matrix and Ingredients	KBC Application and Treatment	Production Method	Major Findings	Ref.
CMC and anthocyanins extract	KBC was used as a filler (1–15 wt%). KBC was cleaned with deionized water and sodium hydroxide (1 M) and dried at 50 °C for 20 h.	Casting method at 40 °C for 18 h.	The incorporation of KBC increased TS from 1.28 to 18.51 MPa and improved UV barrier (200–400 nm) properties in CMC films containing anthocyanin extract. Films incorporated with KBC increased red grapes and plums’ shelf life by up to 25 days.	[11]
PLA plasticized with maleinized linseed oil	KBC was used as a filler (3–5 wt%). KBC was sterilized, cut into small pieces, and dispersed in deionized water in a 1:2 proportion. Dispersions were homogenized in four cycles of 30 s at 30,000 rpm by using an Ultra-turrax. Finally, KBC was dried at 60 °C for 2 days.	Films were produced using a conical twin-screw microextruder. The temperature profile was set at 195–190–190 °C in the three extrusion areas and screw speed was established at 25 rpm. Formulations were mixed during 3 min. The die temperature was set at 180 °C and the film drawing speed at 1200 mm/min. Films with a thickness of 100–200 μm were obtained.	The incorporation of KBC produced a reduction in film transparency and reduced the transmittance in the UV region of the spectra. Furthermore, EM (1308 → 1639 MPa) and TS (13 → 31 MPa) increased with 5 wt% KBC. Unfortunately, WVTR increased from 82 → 116 g/m^2^·day with KBC incorporation.	[19]
KBC	KBC was used as the polymeric matrix. KBC was washed with deionized water (2 × 1.0 L) and pat dried with Kleenex tissues. In sequence, KBC was purified by immersion into a NaOH solution at 90 °C for 1.0 h. Finally, KBC was dried at 50 °C for 20 h to obtain films. Finally, films were modified using citric acid and carbamate groups.	Films were produced by the casting method at room temperature for 24 h.	Citric acid cross-linking resulted in a decrease in TS (25.3 → 7.8 MPa). Whereas carbamate cross-linking with hexamethylene, toluene, methylene di-*p*-phenyl, and 4,4′-methylene-*bis*(cyclohexyl) linking groups by treatments with corresponding diisocyanates resulted improvements in TS (25.3 → 44.1 ± 7.1 MPa), thermal stability (T_onset_ 215 → 281.5 ± 33.5 °C), and reduction in water retention (100 → 60 ± 20%) properties in KBC films.	[20]
Chitosan	KBC was used as a filler (1–3 wt%). No kombucha treatment was reported by the authors.	Casting method at 50 °C for 24 h.	The incorporation of KBC reduced WVP from 256.7 to 132.1 g·mm/cm^2^·h·KPa and enhanced the antioxidant activity (59% DPPH), and the protective effect of the film against ultraviolet. Furthermore, active films reduced lipid oxidation and microbial growth in minced beef during storage.	[21]
Agar and alginate	KBC was used as a filler (2.5 wt%). KBC was cleaned by stirring distilled water for 48 h, filtered and then heated at 50 °C for 12 h with 1 M NaOH, followed by 1 h treatment with 1% glacial acetic acid. KBC was washed with distilled water until the pH reached 7. Finally, KBC was freeze-dried. KBC was treated enzymatically with cellulase.	Casting method at 45 °C for 20 h.	TS of control films (agar and alginate) decreased from 9.98 MPa to 7.69 MPa with the incorporation of unhydrolyzed KBC; however, TS increased to 18.18 MPa when KBC was incorporated into the polymeric matrix. This result was due to the better uniformity and particle size distribution of KBC.	[22]
Alginate and anthocyanins	KBC was used as sn encapsulating material (0.1–0.4 wt%). KBC was ground with a crusher for 4 min at 8000 rpm and then centrifuged at 6000 rpm for 10 min. KBC was hydrolyzed using a 50% (*w*/*v*) sulfuric acid solution in a water bath at 45 °C for 6 h, followed by cleaning with ultra-pure water, centrifugation, and filtering. Hydrolyzed KBC was dialyzed and freeze-dried.	Casting method at 45 °C for 20 h. Oil-in-water (O/W) Pickering emulsions were produced with camelia oil, water, and KBC as an emulsifier, using an ultrasonic dispersion method.	The incorporation of Pickering emulsions containing KBC increased TS from 12 to 33 MPa, reduced transmittance to 280 nm (52 → 3%) and 660 nm (70 → 5%) and increased WCA from 31 to 63°. Films containing Pickering emulsions displayed antioxidant activity.	[23]
KBC	KBC was used as the polymer matrix. KBC was crushed in sterile deionized water and then homogenized at 10,000 rpm by Ultra-Turrax.	N.i.	Materials based on KBC had elongations at a break of 2% and antimicrobial activity against *S. aureus* and *E. coli*.	[24]
KBC, KBC and glycerol or KBC with chitosan	KBC was used as the polymer matrix. KBC was cleaned with NaOH (2 M) at 90 °C for 2 h, and then washed with deionized water 5–6 times. In sequence, KBC was treated with NaClO (2 M) at room temperature for 2 h and finally washed with deionized water for 1 h.	Films were produced by drying KBC with hot air (temperature not provided). Furthermore, KBC was immersed in glycerol of chitosan solutions for 10 min at room temperature, followed by drying to obtain KBC plasticized with glycerol and composite KBC/chitosan films.	The incorporation of glycerol and chitosan increased film thickness (45 → 130 μm), density (6 → 15 g/m^2^), and TS (50 → 110 MPa). KBC, KBC with glycerol, and KBC/chitosan films extended the shelf life of tomatoes by 12, 13, and 15 days when compared with uncoated tomatoes (7 days)	[25]
Gelatin	KBC was used as the encapsulating material (0.1–1 wt%). KBC was cleaned with NaOH (0.1 M) and then washed with distilled water.	O/W Pickering emulsions were produced with cinnamon essential oil and KBC. Gelatin films were produced by the casting method with 1–12% of Pickering emulsions. Films were dried at 25 °C for 48 h.	Gelatin films containing 1% of Pickering emulsion had yellow color, homogeneous visual aspect, and antibacterial activities against *S. aureus* and *E. coli*.	[26]
PLA and PHBV	KBC was used as a filler (5 wt%). KBC was homogenized with distilled water at 25,000 rpm and treated by adding NaOH to the dispersion. The resulting mixture was centrifuged, washed, and freeze dried.	Films were produced by extrusion (twin-screw microextruder) at 180 °C and 100 rpm for 2 min.	Mechanical properties of PLA (EM ≈ 1.7 GPa, TS ≈ 61 MPa, and EB ≈ 4.2%) and PHBV (EM ≈ 2.2 GPa, TS ≈ 31 MPa, and EB ≈ 9.0%) were not altered with the incorporation of KB; however, the film biodegradability increased with the incorporation of KBC. Furthermore, KBC incorporation resulted in a ~23% and ~45% decrease in O_2_ permeability for PLLA and PHBV, respectively.	[27]

CMC: carboxymethyl cellulose; DPPH: 2,2-diphenyl-1-picryl-hydrazyl-hydrate; EM: elastic modulus; N.i.: not informed; PLA: polylactic acid; TS: tensile strength UV: ultraviolet; WVP: water vapor permeability; WVTR: water vapor transmission rate.

## Data Availability

The original contributions presented in the study are included in the article, further inquiries can be directed to the corresponding author.

## Abbreviations

The following abbreviations are used in this manuscript:

AgNPs	Silver nanoparticles
AuNPs	Gold nanoparticles
CMC	Carboxymethyl cellulose
EM	Elastic modulus
PLA	Polylactic acid
PU	Polyurethane
TS	Tensile strength
SDGs	Sustainable Development Goals
WCA	Water contact angle
WVP	Water vapor permeability
WVTR	Water vapor transmission rate

## References

[1] Cavicchia L.O.A., de Almeida M.E.F. (2022). Health Benefits of Kombucha: Drink and Its Biocellulose Production. Braz. J. Pharm. Sci..

[2] Bortolomedi B.M., Paglarini C.S., Brod F.C.A. (2022). Bioactive Compounds in Kombucha: A Review of Substrate Effect and Fermentation Conditions. Food Chem..

[3] Cheng J., Huang S.-Y., Xiong R.-G., Wu S.-X., Yang Z.-J., Zhou D.-D., Saimaiti A., Zhao C.-N., Zhu H.-L., Li H.-B. (2024). Vine Tea Kombucha Ameliorates Non-Alcoholic Fatty Liver Disease in High-Fat Diet Fed Mice via Antioxidation, Anti-Inflammation and Regulation of Gut Microbiota. Food Biosci..

[4] Kitwetcharoen H., Phannarangsee Y., Klanrit P., Thanonkeo S., Tippayawat P., Klanrit P., Klanrit P., Yamada M., Thanonkeo P. (2024). Functional Kombucha Production from Fusions of Black Tea and Indian Gooseberry (*Phyllanthus emblica* L.). Heliyon.

[5] Laavanya D., Shirkole S., Balasubramanian P. (2021). Current Challenges, Applications and Future Perspectives of SCOBY Cellulose of Kombucha Fermentation. J. Clean. Prod..

[6] Chakravorty S., Bhattacharya S., Chatzinotas A., Chakraborty W., Bhattacharya D., Gachhui R. (2016). Kombucha Tea Fermentation: Microbial and Biochemical Dynamics. Int. J. Food Microbiol..

[7] Abuduaibifu A., Tamer C.E. (2019). Evaluation of Physicochemical and Bioaccessibility Properties of Goji Berry Kombucha. J. Food Process Preserv..

[8] Villarreal-Soto S.A., Beaufort S., Bouajila J., Souchard J., Taillandier P. (2018). Understanding Kombucha Tea Fermentation: A Review. J. Food Sci..

[9] Leonarski E., Cesca K., Zanella E., Stambuk B.U., de Oliveira D., Poletto P. (2021). Production of Kombucha-like Beverage and Bacterial Cellulose by Acerola Byproduct as Raw Material. LWT.

[10] Leonarski E., Cesca K., Pinto C.C., González S.Y.G., de Oliveira D., Poletto P. (2022). Bacterial Cellulose Production from Acerola Industrial Waste Using Isolated Kombucha Strain. Cellulose.

[11] El-Shall F.N., Al-Shemy M.T., Dawwam G.E. (2023). Multifunction Smart Nanocomposite Film for Food Packaging Based on Carboxymethyl Cellulose/Kombucha SCOBY/Pomegranate Anthocyanin Pigment. Int. J. Biol. Macromol..

[12] Cottet C., Salvay A.G., Peltzer M.A., Fernández-García M. (2021). Incorporation of Poly(Itaconic Acid) with Quaternized Thiazole Groups on Gelatin-Based Films for Antimicrobial-Active Food Packaging. Polymers.

[13] Cerrutti P., Roldán P., García R.M., Galvagno M.A., Vázquez A., Foresti M.L. (2016). Production of Bacterial Nanocellulose from Wine Industry Residues: Importance of Fermentation Time on Pellicle Characteristics. J. Appl. Polym. Sci..

[14] UN (2025). The 17 Goals. https://sdgs.un.org/goals.

[15] Anukiruthika T., Sethupathy P., Wilson A., Kashampur K., Moses J.A., Anandharamakrishnan C. (2020). Multilayer Packaging: Advances in Preparation Techniques and Emerging Food Applications. Compr. Rev. Food Sci. Food Saf..

[16] Han J.-W., Ruiz-Garcia L., Qian J.-P., Yang X.-T. (2018). Food Packaging: A Comprehensive Review and Future Trends. Compr. Rev. Food Sci. Food Saf..

[17] Dey B., Jayaraman S., Balasubramanian P. (2024). Investigating the Effects of Drying on the Physical Properties of Kombucha Bacterial Cellulose: Kinetic Study and Modeling Approach. J. Clean. Prod..

[18] Ramírez Tapias Y.A., Di Monte M.V., Peltzer M.A., Salvay A.G. (2022). Bacterial Cellulose Films Production by Kombucha Symbiotic Community Cultured on Different Herbal Infusions. Food Chem..

[19] Agüero A., Lascano D., Ivorra-Martinez J., Gómez-Caturla J., Arrieta M.P., Balart R. (2023). Use of Bacterial Cellulose Obtained from Kombucha Fermentation in Spent Coffee Grounds for Active Composites Based on PLA and Maleinized Linseed Oil. Ind. Crops Prod..

[20] Amarasekara A.S., Shrestha A.B., Wang D. (2024). Chemical Modifications of Kombucha SCOBY Bacterial Cellulose Films by Citrate and Carbamate Cross-Linking. Carbohydr. Polym. Technol. Appl..

[21] Ashrafi A., Jokar M., Mohammadi Nafchi A. (2018). Preparation and Characterization of Biocomposite Film Based on Chitosan and Kombucha Tea as Active Food Packaging. Int. J. Biol. Macromol..

[22] He S., Wu Y., Zhang Y., Luo X., Gibson C.T., Gao J., Jellicoe M., Wang H., Young D.J., Raston C.L. (2023). Enhanced Mechanical Strength of Vortex Fluidic Mediated Biomass-Based Biodegradable Films Composed from Agar, Alginate and Kombucha Cellulose Hydrolysates. Int. J. Biol. Macromol..

[23] Li S., Wang X., Luo Y., Chen Z., Yue T., Cai R., Muratkhan M., Zhao Z., Wang Z. (2023). A Green Versatile Packaging Based on Alginate and Anthocyanin via Incorporating Bacterial Cellulose Nanocrystal-Stabilized Camellia Oil Pickering Emulsions. Int. J. Biol. Macromol..

[24] Moghadam F.A.M. (2024). Kombucha Fungus Bio-Coating for Improving Mechanical and Antibacterial Properties of Cellulose Composites. Mater. Today Commun..

[25] Patil S.V., Dulait K., Shirkole S.S., Thorat B.N., Deshmukh S.P. (2024). Dewatering and Drying of Kombucha Bacterial Cellulose for Preparation of Biodegradable Film for Food Packaging. Int. J. Biol. Macromol..

[26] Qin X., Li B., Li L., Wang F., Jia S., Xie Y., Zhong C. (2023). Cinnamon Essential Oil Pickering Emulsions: Stabilization by Bacterial Cellulose Nanofibrils and Applications for Active Packaging Films. Food Biosci..

[27] Koreshkov M., Takatsuna Y., Bismarck A., Fritz I., Reimhult E., Zirbs R. (2024). Sustainable Food Packaging Using Modified Kombucha-Derived Bacterial Cellulose Nanofillers in Biodegradable Polymers. RSC Sustain..

[28] de Farias Nascimento A., Ramos S.M.T., Bergamo V.N., dos Santos Araujo E., Valencia G.A. (2024). Pickering Emulsions Stabilized Using Bacterial Cellulose From Kombucha. Starch—Stärke.

[29] DENG W., LI Y., WU L., CHEN S. (2022). Pickering Emulsions Stabilized by Polysaccharides Particles and Their Applications: A Review. Food Sci. Technol..

[30] Li Z., Hu W., Dong J., Azi F., Xu X., Tu C., Tang S., Dong M. (2023). The Use of Bacterial Cellulose from Kombucha to Produce Curcumin Loaded Pickering Emulsion with Improved Stability and Antioxidant Properties. Food Sci. Hum. Wellness.

[31] Chong A.Q., Chin N.L., Talib R.A., Basha R.K. (2025). Application of Scoby Bacterial Cellulose as Hydrocolloids on Physicochemical, Textural and Sensory Characteristics of Mango Jam. J. Sci. Food Agric..

[32] Ayyappan V.G., Vhatkar S.S., Bose S., Sampath S., Das S.K., Samanta D., Mandal A.B. (2022). Incorporations of Gold, Silver and Carbon Nanomaterials to Kombucha-Derived Bacterial Cellulose: Development of Antibacterial Leather-like Materials. J. Indian Chem. Soc..

[33] Candra A., Ahmed Y.W., Kitaw S.L., Anley B.E., Chen K.-J., Tsai H.-C. (2024). A Green Method for Fabrication of a Biocompatible Gold-Decorated-Bacterial Cellulose Nanocomposite in Spent Coffee Grounds Kombucha: A Sustainable Approach for Augmented Wound Healing. J. Drug Deliv. Sci. Technol..

[34] Muralidharan V., Jebathomas C.R.T., Sundaramoorthy S., Madhan B., Palanivel S. (2024). Preparation and Evaluation of Novel Biodegradable Kombucha Cellulose-Based Multi-Layered Composite Tableware. Ind. Crops Prod..

[35] Nguyen H.T., Saha N., Ngwabebhoh F.A., Zandraa O., Saha T., Saha P. (2023). Silane-Modified Kombucha-Derived Cellulose/Polyurethane/Polylactic Acid Biocomposites for Prospective Application as Leather Alternative. Sustain. Mater. Technol..

[36] Manzocco L., Mikkonen K.S., García-González C.A. (2021). Aerogels as Porous Structures for Food Applications: Smart Ingredients and Novel Packaging Materials. Food Struct..

[37] Bergottini V.M., Bernhardt D. (2023). Bacterial Cellulose Aerogel Enriched in Nanofibers Obtained from Kombucha SCOBY Byproduct. Mater. Today Commun..

[38] Bhattacharyya A., Heo J., Priyajanani J., Kim S.H., Khatun M.R., Nagarajan R., Noh I. (2024). Simultaneous Processing of Both Handheld Biomixing and Biowriting of Kombucha Cultured Pre-Crosslinked Nanocellulose Bioink for Regeneration of Irregular and Multi-Layered Tissue Defects. Int. J. Biol. Macromol..

[39] Mehrotra N., Bhuvana T., Tiwari A., Chandraprakash C. (2024). Aerogel-like Biodegradable Acoustic Foams of Bacterial Cellulose. J. Appl. Polym. Sci..

[40] Rzhepakovsky I., Piskov S., Avanesyan S., Sizonenko M., Timchenko L., Anfinogenova O., Nagdalian A., Blinov A., Denisova E., Kochergin S. (2024). Composite of Bacterial Cellulose and Gelatin: A Versatile Biocompatible Scaffold for Tissue Engineering. Int. J. Biol. Macromol..

[41] Maleki S.S., Mohammadi K., Ji K. (2016). Characterization of Cellulose Synthesis in Plant Cells. Sci. World J..

[42] Heinze T., El Seoud O.A., Koschella A. (2018). Production and Characteristics of Cellulose from Different Sources. Cellulose Derivatives.

[43] Cruz M.A., Flor-Unda O., Avila A., Garcia M.D., Cerda-Mejía L. (2024). Advances in Bacterial Cellulose Production: A Scoping Review. Coatings.

[44] Silva K.A., Uekane T.M., de Miranda J.F., Ruiz L.F., da Motta J.C.B., Silva C.B., Pitangui N. (2021). de S.; Gonzalez, A.G.M.; Fernandes, F.F.; Lima, A.R. Kombucha Beverage from Non-Conventional Edible Plant Infusion and Green Tea: Characterization, Toxicity, Antioxidant Activities and Antimicrobial Properties. Biocatal. Agric. Biotechnol..

[45] Jakubczyk K., Kałduńska J., Kochman J., Janda K. (2020). Chemical Profile and Antioxidant Activity of the Kombucha Beverage Derived from White, Green, Black and Red Tea. Antioxidants.

[46] Jakubczyk K., Łopusiewicz Ł., Kika J., Janda-Milczarek K., Skonieczna-Żydecka K. (2023). Fermented Tea as a Food with Functional Value—Its Microbiological Profile, Antioxidant Potential and Phytochemical Composition. Foods.

[47] Cardoso R.R., Neto R.O., dos Santos D’Almeida C.T., do Nascimento T.P., Pressete C.G., Azevedo L., Martino H.S.D., Cameron L.C., Ferreira M.S.L., Barros F.A.R. (2020). de Kombuchas from Green and Black Teas Have Different Phenolic Profile, Which Impacts Their Antioxidant Capacities, Antibacterial and Antiproliferative Activities. Food Res. Int..

[48] Ramadhan H.U., Prayogo, Kenconojati H., Rahardja B.S., Azhar M.H., Budi D.S. (2021). Potential Utilization of Kombucha as a Feed Supplement in Diets on Growth Performance and Feed Efficiency of Catfish (*Clarias* Sp.). IOP Conf. Ser. Earth Environ. Sci..

[49] Afsharmanesh M., Sadaghi B. (2014). Effects of Dietary Alternatives (Probiotic, Green Tea Powder, and Kombucha Tea) as Antimicrobial Growth Promoters on Growth, Ileal Nutrient Digestibility, Blood Parameters, and Immune Response of Broiler Chickens. Comp. Clin. Path.

[50] Singh P., Tank P.R., Janbandhu S., Motivarash Y. (2022). Effect of supplementation of white button mushroom, *Agaricus bisporus* (IMBACH, 1946) on growth performance and survival in white leg shrimp, *Litopenaeus vannamei* (BOONE, 1931). J. Exp. Zool. India.

[51] Wolfe B.E., Dutton R.J. (2015). Fermented Foods as Experimentally Tractable Microbial Ecosystems. Cell.

[52] Ivanišová E., Meňhartová K., Terentjeva M., Harangozo Ľ., Kántor A., Kačániová M. (2020). The Evaluation of Chemical, Antioxidant, Antimicrobial and Sensory Properties of Kombucha Tea Beverage. J. Food Sci. Technol..

[53] Vīna I., Semjonovs P., Linde R., Deniņa I. (2014). Current Evidence on Physiological Activity and Expected Health Effects of Kombucha Fermented Beverage. J. Med. Food.

[54] Seddiqi H., Oliaei E., Honarkar H., Jin J., Geonzon L.C., Bacabac R.G., Klein-Nulend J. (2021). Cellulose and Its Derivatives: Towards Biomedical Applications. Cellulose.

